# Posible involvement of nitric oxide in anticonvulsant effects of citicoline on pentylenetetrazole and electroshock induced seizures in mice

**DOI:** 10.1016/j.heliyon.2020.e03932

**Published:** 2020-05-19

**Authors:** Rokhsana Rasooli, Fatema Pirsalami, Leila Moezi

**Affiliations:** a Department of Pharmacology, School of Medicine, Shiraz University of Medical Sciences, Shiraz, Iran; b Nanomedicine and Nanobiology Research Center, Shiraz University of Medical Sciences, Shiraz, Iran

**Keywords:** Neuroscience, Citicoline, Seizures, Pentylenetetrazole, Electroshock, Nitric oxide, Mice

## Abstract

Cerebroneurovascular trauma is recognized as an important risk factor in the development of seizure and epilepsy. Administration of citicoline in these situations is a conventional therapeutic strategy, which combines neurovascular protection and repair effects. The aim of the present study is clarifying the effect of acute and sub-chronic citicoline administration on pentylenetetrazole (PTZ) and electroshock induced seizures in mice. Besides we examined the probable role of NO and its interaction with citicoline in seizure experiments.

Male mice were received acute and sub-chronic regimens of different doses of citicoline (62.5, 125, 250 and 500 mg/kg) before the intravenous or intraperitoneal PTZ-induced seizures or electroshock. To clarify the probable role of NO, 7-nitroindazole (7-NI) (60 mg/kg) or aminoguanidine (AG) (100 mg/kg) were injected 5 min before citicoline in separate groups.

The results revealed that neither acute nor sub-chronic treatment with citicoline could affect the seizures induced by intravenous or intraperitoneal PTZ, but in electroshock model, citicoline showed anti-epileptic properties. Co-administration of citicoline and selective nitric oxide synthase (NOS) inhibitors amplified the anticonvulsant effect of citicoline.

The current results indicated that citicoline has anticonvulsant effects probably through the inhibition of NO.

## 1 Introduction

Epilepsy, the most common serious neurological disorder, affecting all age groups in a population and entails major burden of seizure-related disability, mortality and comorbidities [1]. “The WHO's 2010 Global Burden of Disease study ranks epilepsy as the second most burdensome neurologic disorder worldwide in terms of disability-adjusted life years” [2]. The ethiology of the disease is still unknown in about 50% of global cases, however, the underlying cause of epilepsy which can predispose a person to epilepsy may be structural, including a brain injury such as a contusion, intracranial hemorrhage and anoxia in one part of the brain as occurs in stroke or trauma [3]. Subsequent to traumatic brain injury, repair mechanisms would make an imbalance between excitatory and inhibitory potentials, which increases the eventuality of developing spontaneous seizures and epilepsy [4, 5, 6].

Regarding, citicoline is considered as an potential efficient therapeutic agent, which combines neurovascular protection and repair effect, and is prescribed in these situations. Citicoline acts at several levels of the ischemic cascade and its beneficial effects on brain repair have been proved in many documents [7, 8, 9]. Since the neurologists usually prescribe citicoline in cerebroneurovascular complications, so the effect of this drug on the seizure threshold should be evaluated. Exogenously citicoline is a choline donor and source for acetylcholine synthesis, a key neurotransmitter that play critical roles in cellular metabolism [10, 11, 12]. In experimental brain ischemia, the neuroprotective effects of citicoline are attributed to inhibition of glutamate release after ischemia, reducing of free radical generation and probably inhibition of NO production [13].

Nitric oxide (NO) is an intra- and extracellular messenger that mediates diverse signaling pathways in the regulation of multiple cellular processes. Endogenously it is synthesized by NO synthase (NOS) as the product of L-arginine oxidation to L-citrulline, requiring NADPH, molecular oxygen, and a pterin cofactor. Two distinct NOS isoforms exist in mammalian cells; nNOS, eNOS and a third is inducible (iNOS) [14]. Nitric oxide has a critical role in the mechanisms underlying seizure initiation and/or propagation. A large body of in vivo and in vitro research have shown that NO provides controversial role in epipepsy with either anti-convulsant or pro-convulsant effects [15, 16, 17, 18, 19, 20].

To clarify the effects of citicoline on seizure threshold, here we used three animal models of intravenous (IV) and intraperitoneal (IP) PTZ, and electroshock (ECT) in mice. Additionally the probable interaction of citicoline and NO in convulsion is investigated.

## 2 Materials and methods

### 2.1 Drugs

The following drugs were used: Citicoline (Alborz Daru, Iran), PTZ (Sigma, UK), aminoguanidine (a selective iNOS inhibitor) (Sigma, USA), and 7-nitroindazole (7-NI) (a selective nNOS inhibitor) (Sigma, Canada). Citicoline, AG, and PTZ were dissolved in physiological saline. 7-NI was suspended in a 1% aqueous solution of DMSO. All injections were administered in a volume of 10 mL/kg. Appropriate vehicle controls were run for each experiment. In all experiments, citicoline, AG and 7-NI were administered intraperitoneally. To assess clonic seizure threshold and latency, PTZ was administered intravenous and intraperitoneal, respectively.

### 2.2 Animals

Adult male albino mice (Laboratory animal center, Shiraz University of Medical Sciences, Shiraz, Iran) aged 6–8 weeks and weighing 22–28 g were used throughout this study. Animals were housed under standard laboratory conditions that included controlled ambient temperature (21 ± 2 °C), a 12-hour-dark/12-hour-light (7:00 a.m. to 7:00 p.m.) cycle, and free access to food (standard mouse chow pellets) and water. The animals were housed in groups of 5 per cage. The animals were randomly distributed into different groups. Each mouse was used only once, and by average each treatment group consisted of 8–10 animals. All the procedures were carried out in accordance with institutional guidelines for animal care and use. The procedure approved by Shiraz University of Medical Sciences Ethics Committee prior to conducting our research (No: 95-01-36-13167).

### 2.3 Seizure models

#### 2.3.1 Intraperitoneal PTZ-induced seizure

We examined the effects of different treatment groups on generalized tonic–clonic seizures induced by intraperitoneal injection of high dose PTZ. Generalized tonic-clonic seizure induced by intraperitoneal (IP) PTZ is a distinct model related to grand mal seizures in humans [21]. In this model, PTZ (85 mg/kg, CD97 for clonic seizures in the current experiment) was administered with a single intraperitoneal injection to evaluate the latency for the onset of clonic seizures and the incidence of tonic and death following seizures [22]. Immediately after injection of PTZ, animals were transferred to an open field (50 cm in diameter) and monitored for the appearance of convulsion or death for 30 min. Following the administration of 85 mg/kg of PTZ time latencies for myoclonic and clonic seizures were measured. Latency was defined as the time between PTZ injections and the onset of seizures. The incidence of tonic seizure and death was also recorded.

#### 2.3.2 Intravenous PTZ-induced seizure

An intravenous (IV) PTZ infusion with a constant flow rate through the tail vein in mice elicits seizure response in a reliable, reproducible and rapid manner. The test is based on threshold dose of PTZ to induce seizures in contrast to threshold time to induce seizures in fixed dose in IP PTZ paradigm [23]. The clonic seizure threshold was determined by inserting a 30-gauge dental needle into the lateral tail vein of the mouse [24,25]. The needle was then secured to the tail with a narrow piece of adhesive tape. With the mouse moving freely, the PTZ solution (0.5%)was infused into the tail vein at a constant rate of 0.5 ml/min using an infusion pump (Harvard, USA), which was connected to the dental needle by polyethylene tubing. Infusion was halted when forelimb clonus followed by full clonus of the body was observed. The minimum dose of PTZ (mg/kg mouse wt) to induce a clonic seizure, was measured as an index of clonic seizure threshold.

#### 2.3.3 Electroshock test

To examine the effect of citicoline in modulation of susceptibility to ECT-induced seizure [26, 27, 28], an electroconvulsive therapy apparatus (Model 7800, Ugo Basile, Camerio, Italy) was applied. Tonic convulsions of the hind extremities of mice were induced by passing alternating current (50 Hz, 50 mA and 0.2 s) via ear electrodes. In order to improve electrode contact, the electrodes were moistened with normal saline before being attached to the ears of mice. Electroshock induces a continuum of motor convulsions that are dependent on the intensity of the electrical stimulation current. The current used was predetermined before experimentation and was the current that caused hind limb extension in all mice in the trials. Data were expressed as the duration of tonic hind-limb extension (THE) after ECT.

### 2.4 Study design

#### 2.4.1 Short time (acute) treatment with different doses of citicoline on intravenous (IV) and intraperitoneal (IP) PTZ and electroshock (ECT) seizure models of mice

The animals were divided into 4 groups received a single injection of different doses of citicoline (0 [saline], 125, 250 and 500 mg/kg, IP citicoline injection) 60 min before evaluation of the IV or IP PTZ-induced seizures or ECT. Selecting the dose of citicoline was according to clinical trial in patients and experimental researches [13,29,30].

#### 2.4.2 Long time (sub-chronic) treatment with different doses of citicoline on IV and IP PTZ and ECT seizure models of mice

In long time pretreatment, similarly, the animals were divided into 4 groups, as follows: (0 [saline], 125, 250 and 500 mg/kg, IP citicoline injection). In ECT group we had five groups including 4 groups like above and a group which received citicoline (62.5 mg/kg). The animals were injected by citicoline or saline for 6 days. In the 7^th^ day, mice received the same doses of citicoline or solvent 60 min before evaluation of the IV or IP PTZ-induced seizure or ECT.

#### 2.4.3 Acute and sub-chronic treatment with citicoline in the presence of AG or 7-NI in ECT-induced seizure model

In acute experiments, AG (a selective iNOS inhibitor) (100 mg/kg) or 7-NI (a selectivenNOS inhibitor) (60 mg/kg) was administered 5 min before citicoline (0 and 62.5 mg/kg) and 65 min before electroshock.

In sub-chronic experiments, the animals were injected by citicoline or saline for 6 days. In the 7^th^ day, AG (100 mg/kg) or 7-NI (60 mg/kg) was administered [31]5 min before citicoline (0 and 62.5 mg/kg) and 65 min before electroshock.

### 2.5 Statistical analysis

Data are expressed as mean ± SEM. Fisher's exact probability test, as well as analysis of variance (ANOVA) were used for statistical evaluation. The p-values less than 0.05 were considered to be statistically significant.

## 3 Results

### 3.1 Effect of acute and sub-chronic treatment with different doses of citicoline on the seizure threshold in IV PTZ modelof mice

**Figure 1:**
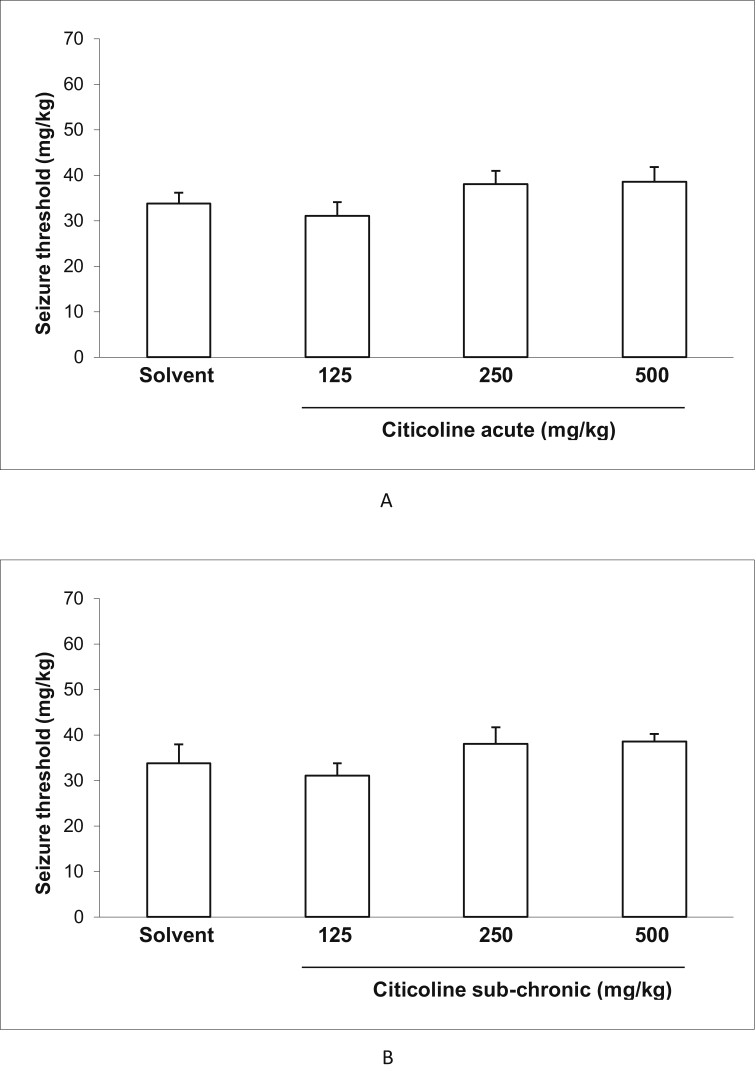
Effect of acute (A) and sub-chronic (B) treatment with different doses (125, 250 and 500 mg/Kg) of citicoline on the seizure thresholdin IV PTZ model. Data are presented as mean ± S.E.M.

As depicted in Figure 1, citicoline neither in acute nor in sub-chronic treatment with different doses show notable effect on the seizure threshold in IV PTZ model.

### 3.2 Effect of acute and sub-chronic treatment with different doses of citicoline on the latency of myoclonic and clonic seizuresand incidence of tonic seizure and death in IP PTZ seizure model of mice

**Figure 2:**
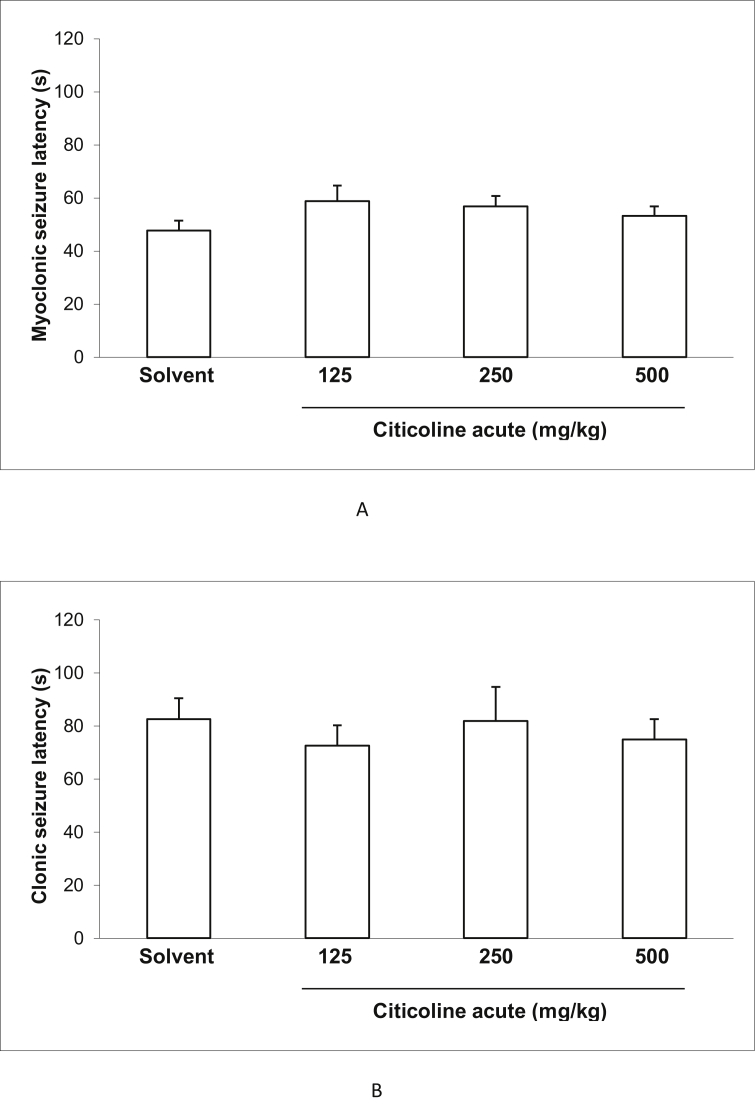
Effect of acute treatment with different doses (125, 250 and 500 mg/Kg) of citicoline on myoclonic seizure latency (A) and clonic seizure latency (B) in IP PTZ model. Data are presented as mean ± S.E.M.

**Figure 3:**
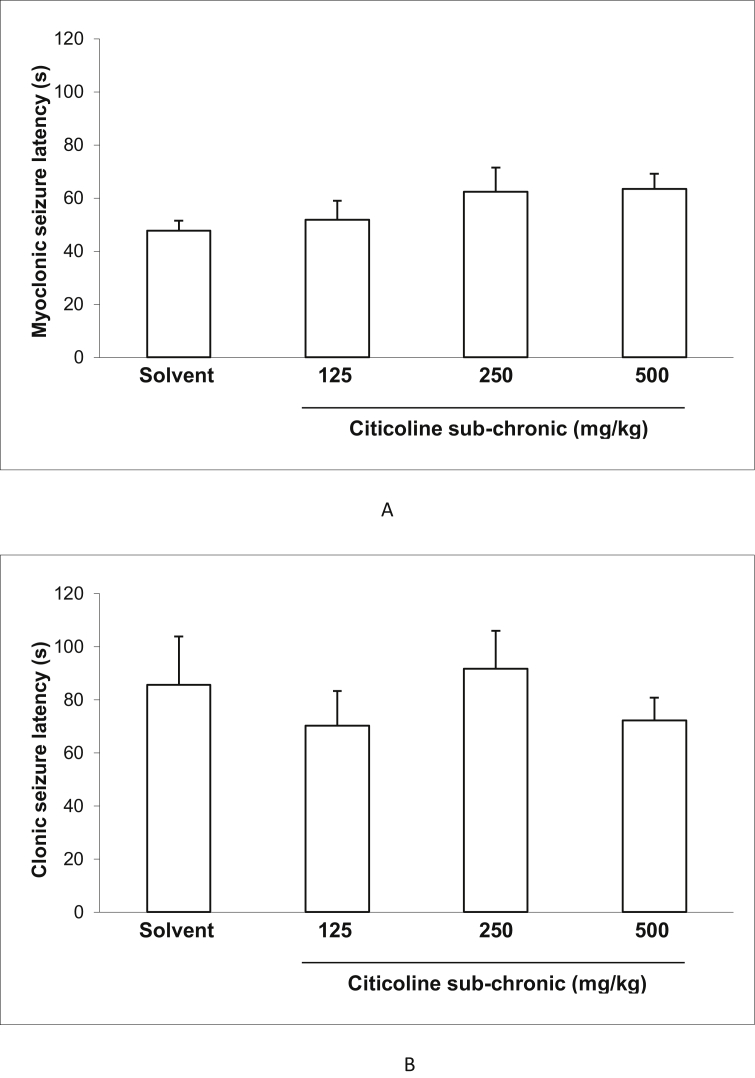
Effect of sub-chronic treatment with different doses (125, 250 and 500 mg/Kg) of citicoline on myoclonic seizure latency (A) and clonic seizure latency (B) in IP PTZ model. Data are presented as mean ± S.E.M.

Figures 2 and 3 show the effect of acute and sub-chronic administration of different doses of citicoline (125, 250and500 mg/kg) on the latency of myoclonic and clonic seizures. There were no significant effects between the latency of myoclonic and clonic seizures at all doses of citicoline compared to the control mice in both acute and sub-chronic treatments.

As demonstrated in Tables 1 and 2, acute administration of none of the doses of citicoline could protect from tonic seizure or prevent from mortality subsequent to intraperitoneal pentylenetetrazole injection. Besides, sub-chronic administration of citicoline did not also show significant effect on the incidence of tonic seizure and death.

**Table 1:** Effect of acute treatment with different doses of citicoline on the incidence of tonic seizure and death in intraperitoneal PTZ-induced seizure model.

Groups	Tonic seizure protection (%)	Mortality protection (%)
Solvent	45.5	54.5
Citicoline (125 mg/kg)	50	50
Citicoline (250 mg/kg)	44.4	55.6
Citicoline (500 mg/kg)	50	50

Percentage of protection against incidence of tonic seizures and death subsequent pentylenetetrazole was compared among groups using Fisher's exact test (P > 0.05 for both tonic seizure protection and mortality protection). Each group of mice consisted of eight to ten animals.

**Table 2:** Effect of sub-chronic treatment with different doses of citicoline on the incidence of tonic seizure and death in intraperitoneal PTZ-induced seizure model.

Groups	Tonic seizure protection (%)	Mortality protection (%)
Solvent	45.5	54.5
Citicoline (125 mg/kg)	50	62.5
Citicoline (250 mg/kg)	57.1	57.1
Citicoline (500 mg/kg)	57.1	57.1

Percentage of protection against incidence of tonic seizures and death subsequent pentylenetetrazole was compared among groups using Fisher's exact test (P > 0.05 for both tonic seizure protection and mortality protection). Each group of mice consisted of eight to ten animals.

### 3.3 Effect of acute and sub-chronic treatment with different doses of citicoline on the duration in electroshockmodel

**Figure 4:**
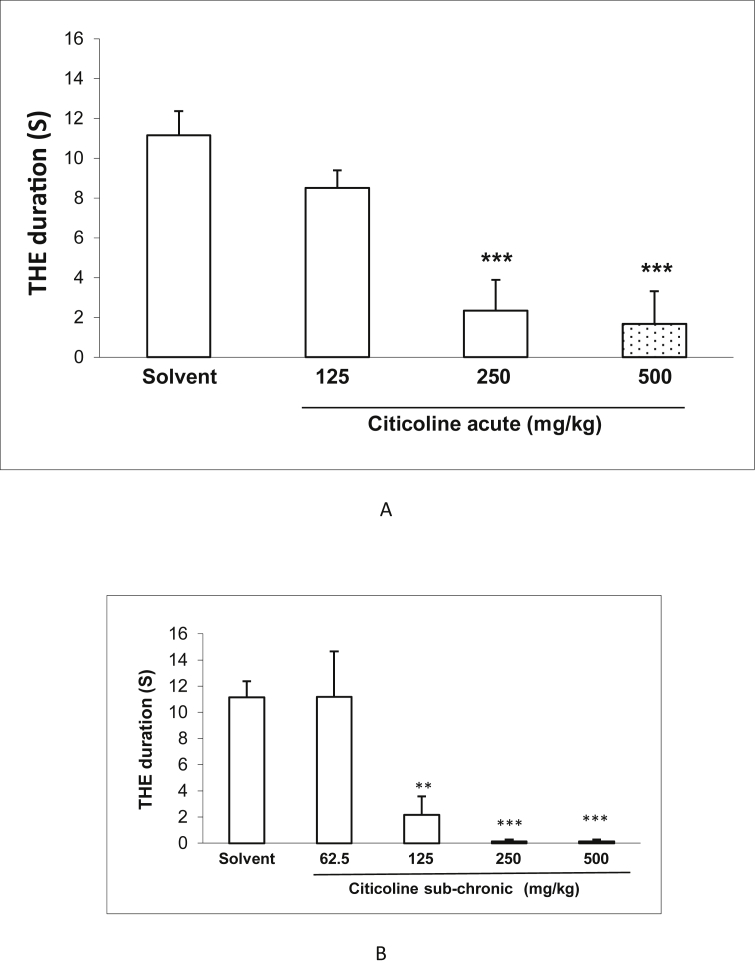
Effect of acute and sub-chronic treatment with different doses (125, 250 and 500 mg/Kg) of citicoline on the duration of tonic hind-limb extension (THE). Data are presented as mean ± S.E.M. ∗∗p < 0.01 and ∗∗∗p < 0.001 compared with physiological saline group.

Figure 4 shows the effect of acute and sub-chronic administration of different doses of citicoline (125, 250 and 500 mg/kg) on ECT-induced seizure. Compared to control group, citicoline in acute administration at the doses of 250 and 500 significantly decreased the duration of seizure (*P* < 0.001). In sub-chronic administration, besides the doses of 250 and 500 mg/kg (*P* < 0.001), the dose of 125 mg/kg could also decreased the duration of seizure, significantly (*P* < 0.01).

### 3.4 Effect of acute and sub-chronic treatment with citicoline in the presence of AG or 7-NI on the duration in electroshock model

**Figure 5:**
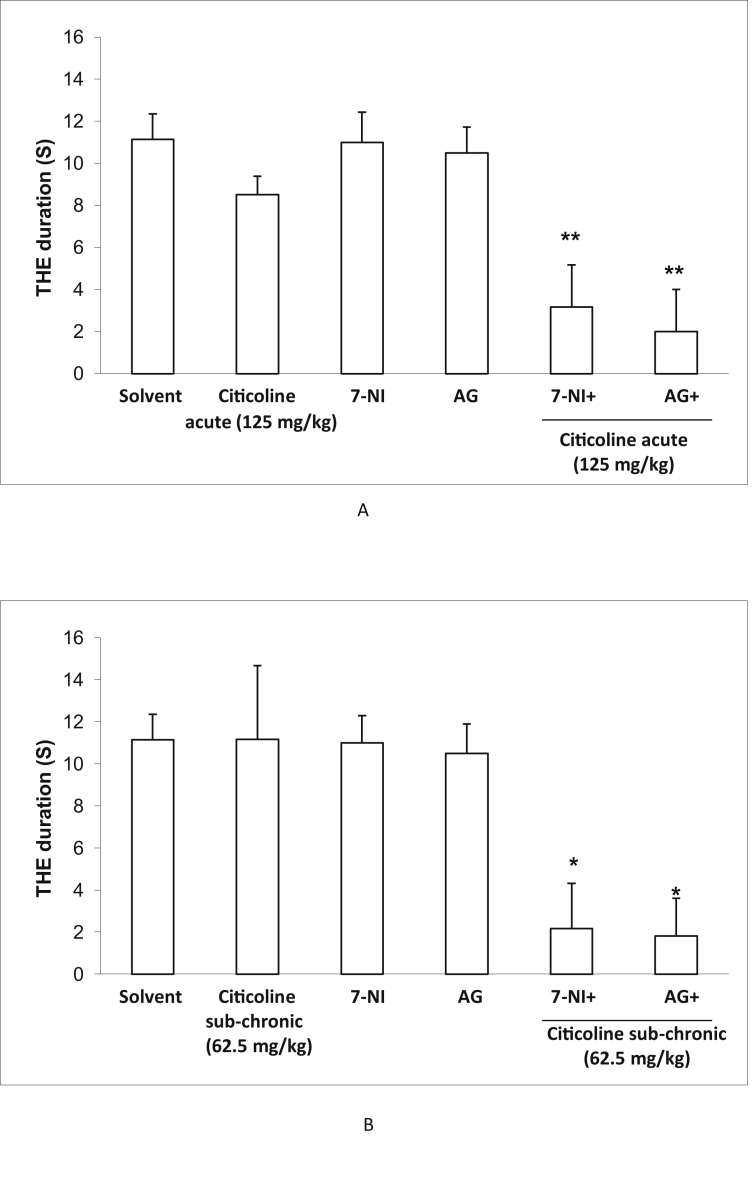
Effect of acute and sub-chronic treatment with 62.5 mg/kg citicoline in the presence of aminoguanidine (a selective iNOS inhibitor) (AG) or 7-nitroindazole (a selective nNOS inhibitor) (7-NI) on tonic hind-limb extension (THE) duration in electroshock model. Data are expressed as mean ± SEM. ∗p < 0.05 and ∗∗p < 0.01 compared with physiological saline group.

To investigate the probable role of nitric oxide in citicoline-induced anticonvulsant effects, we administered AG, a selective inhibitor of iNOS or 7-NI a selective inhibitor of nNOS and eNOS, with and without ineffective dose of citicoline in both acute and sub-chronic regimens in electroshock model. As depicted in Figure 5, when ineffective dose of citicoline was co-administrated with AG or 7-NI, anticonvulsant effect was observed, while each treatment separately did not affect the duration of seizure.

## 4 Discussion

The results of the present study revealed that neither acute nor sub-chronic treatment by citicoline could affect the seizures induced by i.p or i.v PTZ; however in electroshock model, opposite findings were observed. In electroshock model, citicoline in both acute and sub-chronic administration routes decreased the duration of THE. There are controversies over the anticonvulsant or proconvulsant features of citicoline. Previous studies showed the protective role of choline intake in models of pilocarpine and kainic acid evoked epilepsy [32]. Abdolmaleki et al. have reported the anticonvulsant effect of citicoline in the PTZ seizure model [33]. Karapova et al. also investigated the inhibitory effects of citicoline on acute PTZ-induced generalized epileptiform activity using a mouse model. Their results showed that citicoline at higher doses has an anticonvulsive effect on acute generalized seizure activity [34]. In contrast, the study of Kim et al. on the pilocarpine-induced seizure model, not only showed no protective effect of citicoline treatment, but also proved the adversely effects of citicoline on seizure [35].

Our findings in PTZ model were in contrast with all previous evidence and we observed no desired or no adverse effects in the mice, however the protective effect of citicoline was seen in ECT model.

To clarify the probable role of NO in citicoline anticonvulsant effect in electroshock model, the effect of acute and sub-chronic treatment with ineffective dose of citicoline in the presence of AG (a selective inhibitor of iNOS) or 7-NI (a selective inhibitor of nNOS) on the seizure duration was examined. In the groups received acute and sub-chronic ineffective dose of citicoline, treatment of mice with non-effective doses of AG, exerted anticonvulsant effect in electroshock model. This effect was also observed in co-administration of ineffective dose of citicoline and 7-NI. Amplifying the anti-covulsant effect, suggests that citicoline and AG or 7-NI act in a similar pathway. As both AG or 7-NI, are selective inhibitors of NOS enzyme, it is possible that citicoline partly exerts its anti convulsant effect via inhibition of NO production. In agreement with our findings, Park et al, showed that citicoline could partly exerts its neurologiacal effects via decreasing of NOS isoforms expression and decreasing of NO production [36]. NO is synthesized from oxidation of the amino acid l-arginine by nitric oxide synthase (NOS) including endothelial NOS (eNOS), inducible NOS (iNOS) and neuronal NOS (nNOS) and acts as a neuromodulator of neurotransmitters in the brain [37] and has been shown as a modulator of seizure susceptibility with diverse anticonvulsant or proconvulsant effects in different seizure models. Both NOS isoforms including eNOS and iNOS may be implicated in the development of seizures [38, 39, 40, 41]. It seems that model dependent effects of citicoline probably is due to various effects of NO in different models. It has been suggested that NO synthesized at the postsynaptic level by activation of postsynaptic N-methyl-D-aspartate (NMDA) receptors. NMDA receptors have a prominent role in the pathogenesis of neuronal death and epilepsy, as a convulsant [42,43]. Citicoline is shown to antagonize NMDA receptors in the brain and therefore inhibit NO production [44,45]. Mir et al demonstrated that citicoline prevents glutamate mediated neuronal cell death in cerebellar granule cells [46]. Here, it seems that citicoline probably could exert its anti-seizure effects via prevention of destructive effects of NO. Many experimental studies have demonstrated that citicoline reduces neurodegeneration induced by ischemia, hypoxia, traumatic brain injury, and β-amyloid depositions in animals [30,46].

Considering these features, the protective role of citicoline in seizure and convulsion become justifiable.

Taken together the present results indicated that administration of citicoline exerted anti-convulsant effects in electroshock model of seizure. We also pointed out for the first time that inhibition of nitric oxide production through iNOS and nNOS inhibitors might involve in citicoline-induced anticonvulsant effects.

## Declarations

### Author contribution statement

Rokhsana Rasooli: Conceived and designed the experiments; Performed the experiments; Contributed reagents, materials, analysis tools or data; Wrote the paper.

Fatema Pirsalami: Performed the experiments; Contributed reagents, materials, analysis tools or data.

Leila Moezi: Conceived and designed the experiments; Analyzed and interpreted the data; Contributed reagents, materials, analysis tools or data; Wrote the paper.

### Funding statement

This work was supported by 10.13039/501100004320Shiraz University of Medical Sciences grants No: 95-01-57-11482.

### Competing interest statement

The authors declare no conflict of interest.

### Additional information

No additional information is available for this paper.
